# Activation of the Ubiquitin Proteasome Pathway by Silk Fibroin Modified Chitosan Nanoparticles in Hepatic Cancer Cells

**DOI:** 10.3390/ijms16011657

**Published:** 2015-01-12

**Authors:** Ming-Hui Yang, Tze-Wen Chung, Yi-Shan Lu, Yi-Ling Chen, Wan-Chi Tsai, Shiang-Bin Jong, Shyng-Shiou Yuan, Pao-Chi Liao, Po-Chiao Lin, Yu-Chang Tyan

**Affiliations:** 1 Department of Medical Research, Kaohsiung Medical University Hospital, Kaohsiung 807, Taiwan; E-Mails: myang@mail.kmuh.org.tw (M.-H.Y.); yuanssf@kmu.edu.tw (S.-S.Y.); 2 Translational Research Center, Kaohsiung Medical University Hospital, Kaohsiung 807, Taiwan; 3 Department of Biomedical Engineering, National Yang-Ming University, Taipei 112, Taiwan; E-Mail: twchung@ms.ym.edu.tw; 4 Department of Medical Imaging and Radiological Sciences, Kaohsiung Medical University, Kaohsiung 807, Taiwan; E-Mails: eshan5413@gmail.com (Y.-S.L.); jongsb@kmu.edu.tw (S.-B.J.); 5 Department of Nuclear Medicine, Kaohsiung Medical University Hospital, Kaohsiung 807, Taiwan; E-Mail: 1010.yiling@gmail.com; 6 Department of Medical Laboratory Science and Biotechnology, Kaohsiung Medical University, Kaohsiung 807, Taiwan; E-Mail: wanchi@cc.kmu.edu.tw; 7 Department of Laboratory Medicine, Kaohsiung Medical University Hospital, Kaohsiung 807, Taiwan; 8 Department of Obstetrics and Gynecology, Kaohsiung Medical University Hospital, Kaohsiung 807, Taiwan; 9 School of Medicine, College of Medicine, Kaohsiung Medical University, Kaohsiung 807, Taiwan; 10 Department of Environmental and Occupational Health, National Cheng Kung University, Tainan 701, Taiwan; E-Mail: liaopc@mail.ncku.edu.tw; 11 Department of Chemistry, National Sun Yat-sen University, Kaohsiung 804, Taiwan; E-Mail: pclin@mail.nsysu.edu.tw; 12 National Sun Yat-sen University–Kaohsiung Medical University Joint Research Center, Kaohsiung 804, Taiwan; 13 Institute of Medical Science and Techmology, National Sun Yat-sen University, Kaohsiung 804, Taiwan

**Keywords:** silk fibroin, chitosan nanoparticle, biomaterial, proteomics, ubiquitin proteasome pathway

## Abstract

Silk fibroin (SF) is a protein with bulky hydrophobic domains and can be easily purified as sericin-free silk-based biomaterial. Silk fibroin modified chitosan nanoparticle (SF-CSNP), a biocompatible material, has been widely used as a potential drug delivery system. Our current investigation studied the bio-effects of the SF-CSNP uptake by liver cells. In this experiment, the characterizations of SF-CSNPs were measured by particle size analysis and protein assay. The average size of the SF-CSNP was 311.9 ± 10.7 nm, and the average zeta potential was +13.33 ± 0.3 mV. The SF coating on the SF-CSNP was 6.27 ± 0.17 μg/mL. Moreover, using proteomic approaches, several proteins involved in the ubiquitin proteasome pathway were identified by analysis of differential protein expressions of HepG2 cell uptake the SF-CSNP. Our experimental results have demonstrated that the SF-CSNP may be involved in liver cancer cell survival and proliferation.

## 1. Introduction

Silk fibroin (SF) is an insoluble protein with bulky hydrophobic domains, which consists of layers of antiparallel β sheets, and can be easily purified as sericin-free silk-based biomaterials [1]. It is emitted from silkworms and spiders, the larvae of *Bombyx mandarins* and *Bombyx mori*, other moth genera such as *Antheraea*, *Cricula*, *Samia* and *Gonometa*, and numerous other insects. The primary structure of silk fibroin is characterized as natural block copolymers comprising hydrophobic blocks with a repetitive sequence of short side-chain amino acids such as glycine and alanine, as well as mainly consisting of the recurrent amino acid sequence (Gly–Ser–Gly–Ala–Gly–Ala)*_n_*. The raw state consists of two main proteins, sericin and fibroin, with a glue-like layer of sericin coating two singular filaments of fibroin called brins [2,3]. Such material is highly applicable due to its low immune or inflammatory response and favorable biological response characteristics [4]. SF-based biomaterials have been investigated in the form of files, fibers, hydrogels, particles and scaffolds [5,6,7,8], and in applications of vascular, neural, skin, bone and cartilage tissue regeneration [9,10,11,12]. Increasingly, SF is exploited in other areas of biomedical science, as a result of new knowledge of its processing and properties like mechanical strength, elasticity, biocompatibility, and controllable biodegradability [13]. These properties of SF are particularly useful for tissue engineering.

Biomaterials play important roles in regenerative medicine, tissue engineering and drug delivery [14]. Nanomedicine is the application of nanotechnology in medicine, which has enabled the development of nanoparticle therapeutic carriers. Nanomaterials have increased surface to volume ratio compared with their bulk materials, and this may confer interesting properties, such as increased mechanical strength. Their distinct physicochemical characteristics, obtained by changing the size and shape, are very different from their natural materials and thus grant new possibilities [15]. The development of nanoparticulate drug carriers has followed several routes depending on the final application. Although a wide variety of systems have been designed with their own advantages and limitations, the common goal is to rationalize drug delivery to enhance the bioavailability of the drugs towards targeted diseased cells, promoting the required response while minimizing side-effects. Drug delivery to tumors is exacerbated by the toxicity to normal cells in conjunction with low absorption at the tumor-site due to low retention of drugs by the tumor cells [16]. However, cell proliferation, differentiation and regeneration of tissues all depend upon the interactions between biomaterial surfaces and cells.

The tumor microenvironment includes surrounding blood vessels, cells, signaling molecules, and the extracellular matrix (ECM) [17]. Most tumor types (~85%) are carcinomas, or cancers in the epithelial tissue. Usually, these kinds of tumors are not vascularized, which prevent the tumor sizes from growing more than 2 mm without inducing new blood vessels to feed themselves [18]. To feed the tumor cells, angiogenesis is dysregulated and as a result the vasculature formed differs from that of normal tissue. Using a nanocarrier is now considered the gold standard of targeted cancer therapy and used as a transport module for drugs which can pass through the vasculature. Nanocarrier vehicles (~20–300 nm in diameter) have been developed for drug and other therapeutic molecule transportation, and these therapies can be targeted to selectively extravasate through the tumor vasculature via the enhanced permeability and retention (EPR) effect. Common nanomaterials used for nanocarriers include micelles, carbon nanotubes, liposomes and other substances. However, since some important normal tissues such as the liver and kidneys, also have fenestrated endothelium, great care must be taken with using the correct size (10–300 nm, with greater retention in tumors seen in using larger nanocarriers) and charge (anionic or neutral) [19].

The silk fibroin modified chitosan nanoparticles (SF-CSNPs) have continued to gain more attention as drug delivery carriers because of their better stability, low toxicity, simple and mild preparation methods, and because they provide versatile routes of administration [20]. The SF-CSNPs are targeted to tumor cell surfaces through the enhanced permeability and retention effect; thus, they are very suitable for chemotherapeutic delivery in cancer treatment. The accumulation of SF-CSNPs typically occurs around the defect area in cells and tissues by hydrophobic interactions. In addition, several physiochemical characteristics of SF-CSNPs, such as ability to cross biological barriers, to protect macromolecules from degradation, and to deliver a compound at a target site, need to be determined as favorable [21]. Therefore, to use SF derivatives for biomedical applications, a test for evaluating biocompatibility must be performed.

“Proteome” and “proteomics” are relatively new words, coined by Wilkins *et al.*, in 1994 [22]. The proteome is the entire set of proteins expressed by the genome. Proteomic analysis means a comprehensive analysis of proteins, and proteomics is the science by which proteins are comprehensively investigated with regard to their roles as functional elements. Recently, characterization of these cellular proteins by proteomic approaches has revealed that the surface of biomaterials defines the protein reactivity and the protein-biomaterial interaction.

In this study, we investigated various methods to analyze and characterize the parameters that influence the uptake of cells on SF-CSNP. The CCL-13 and HepG2 cells served as cell models for the uptake of SF-CSNPs. For instance, using colorimetric techniques such as the lactate dehydrogenase (LDH) and Bromodeoxyuridine (5-bromo-2'-deoxyuridine, BrdU) assays are convenient methods and are typically applied to much of the current biomaterial research. Characterizations of SF-CSNPs were observed by particle size and zeta potential analysis. To evaluate early responses of CCL-13 and HepG2 cells to SF-CSNPs, a mass spectrometry-based proteomic profiling system was adopted for assessing characteristic proteins that were expressed due to the interactions of CCL-13 and HepG2 cells with SF-CSNPs. Through the investigation, various proteins that influence the early responses of CCL-13 and HepG2 cells on SF-CSNP were found; and, as far as we know, some have not yet been reported in the study of cell-nanoparticle interactions.

## 2. Results and Discussions

### 2.1. Size, Zeta Potential, and Morphology of Silk Fibroin Modified Chitosan Nanoparticles (SF-CSNPs)

In this study, the CSNP was used as the base material and the SF was adsorbed onto the surface of CSNP. The SF-CSNPs were washed and isolated by ultracentrifugation (25 kg, 15 min) and then re-suspended in distilled deionized water (1 mL) by manual shaking. The protein concentrations of the SF-CSNPs suspension were measured by Bio-Rad Bradford total protein assay kit (Bio-Rad Laboratories, Inc. Hercules, CA, USA) with a mean of 6.27 ± 0.17 μg/mL. The result of protein assay for the CSNP suspension was not detected. As determined by particle size and zeta potential analyzers, the average size of the SF-CSNP was 311.9 ± 10.7 nm with the morphology of a spherical micelle, and the average zeta potential was +13.33 ± 0.3 mV in phosphate-buffered saline. The particle size of SF-CSNP was larger than CSNP, due to the SF adsorbed onto the CSNP surface. The surface charge of nanoparticles, also called zeta potential, is a concept used to describe the electrokinetic properties of a colloidal particle under the influence of an applied electric field, which can influence the nanoparticle stability in suspension through the electrostatic repulsion between nanoparticles. A larger zeta potential value means the particles are more stable and less likely to form aggregates. In our study, the surface charge of CSNPs was positive, resulting from the protonation of NH_2_ functional groups of glucosamine units to NH_3_^+^ ion. After the SF adsorbed onto the CSNP surface, the zeta potential was neutralized by the functional groups (COO^−^) of SF, and the SF-CSNPs were formed by layers self-assembled on CSNPs. The zeta potential of CSNP was around 15.13 mV, so the negatively charged SF molecules promoted the self-assembly with positively charged CSNPs. The zeta potential of the SF-CSNPs decreased significantly by approximately 2 mV (around −11.9%). Thus, the electrostatic forces can promote the aggregation of SF molecules and consequently, it is expected that the SF may easily self-assemble on CSNP surfaces. The conjugation of surface-modified SF with CSNPs has shown an average zeta potential of 13.33 mV, which demonstrates that the surface of CSNPs was well modified with SF. The data is shown in Table 1.

**Table 1:** Characterization of nanoparticles.

Nanoparticle	Concentration (μg/mL)	Particle Size (nm) #	Zeta Potential (mV) #
CSNP	N.D.	239.3 ± 7.3	15.13 ± 0.6
CSNP coating SF	6.27 ± 0.17 **	311.9 ± 10.7 **	13.33 ± 0.3 *

# The particle size and zeta potential of these nanoparticles were determined in phosphate-buffered saline (pH = 7.4); Data are expressed as mean ± standard error, *n* = 6, * *p* < 0.05, ** *p* < 0.001, (*t*-test); N.D.: Not detected.

### 2.2. In Vitro SF-CSNP Uptake

**Figure 1:**
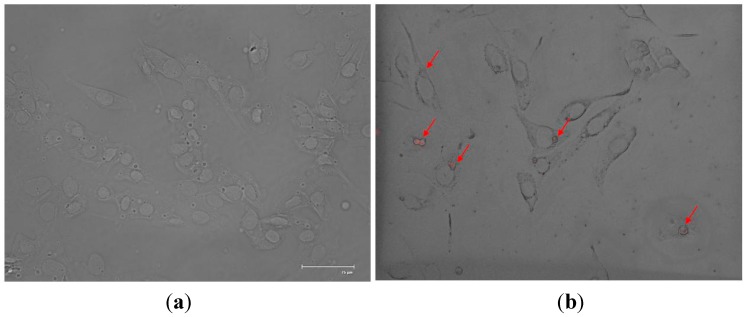
The live fluorescent images of silk fibroin modified chitosan nanoparticles (SF-CSNPs) (red) taken by HepG2 cells. (**a**) cells without SF-CSNPs; (**b**) cells with SF-CSNPs at a concentration of 5 μg/mL for 12 h at 37 °C, the red fluorescence was localized near the cell nuclei. Images represent merged images of differential interference contrast (DIC) and red fluorescence. (600×, scale bar: 75 μm).

The *in vitro* uptake of SF-CSNPs was evaluated by fluorescence microscopy. The HepG2 cell was incubated with the growth medium containing SF-CSNPs at a concentration of 5 μg/mL for 12 h at 37 °C. As expected, no red fluorescence signals were detected in sections of the cells without SF-CSNPs (Figure 1a, 600×, scale bar: 75 μm). Cell micrographs from HepG2 cells treated with SF-CSNPs revealed red fluorescence localized near the cell nuclei and within the cytoplasm (Figure 1b, 600×, red arrows).

### 2.3. Cytotoxicity of SF-CSNP

Cell death is assayed by the quantification of a stable cytoplasmic enzyme activity, LDH, which is released into the cell culture supernatant upon cell death and damage of the cytoplasmic membrane. BrdU is integrated into newly synthesized DNA strands of actively proliferating cells. Following partial denaturation of double stranded DNA, BrdU is detected immunochemically allowing the assessment of the number of cells which are synthesizing DNA. To examine the cytotoxicity, HepG2 and CCL-13 cells were incubated with CSNPs and SF-CSNPs for 12 h. LDH and BrdU assays are quantitative colorimetric assays for mammalian cell survival and cell proliferation.

**Figure 2:**
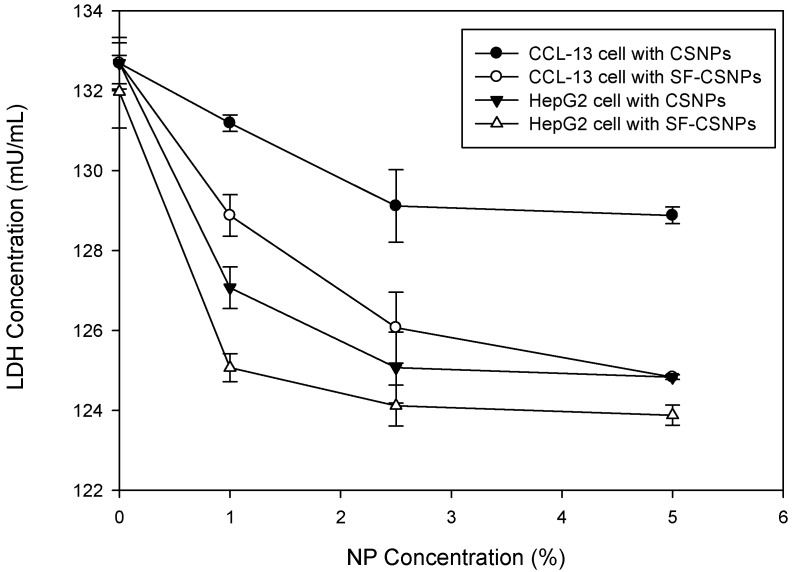
Lactate dehydrogenase (LDH) test of SF-CSNP and CSNP effects on CCL-13 and HepG2 cells (*n* = 6, mean ± standard error, *t*-test, *p* < 0.05).

**Figure 3:**
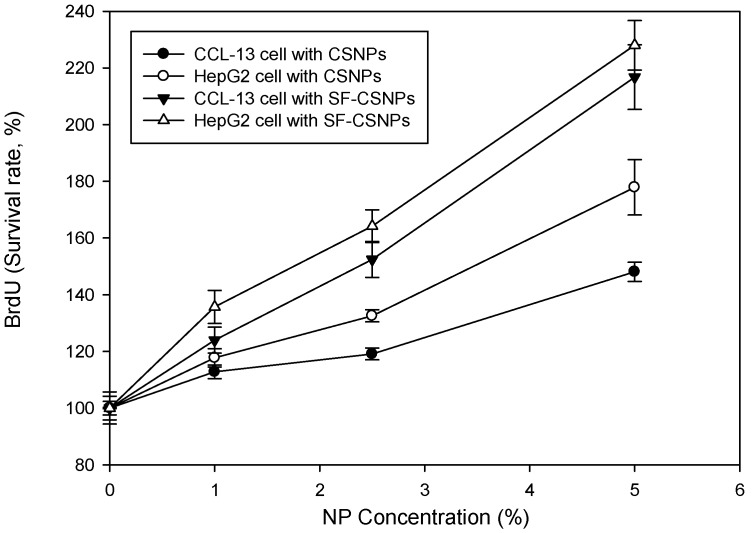
Proliferation (Bromodeoxyuridine (5-bromo-2'-deoxyuridine, BrdU)) test of SF-CSNP and CSNP effects on CCL-13 and HepG2 cells (*n* = 6, mean ± standard error, *t*-test, *p* < 0.05).

As shown in Figure 2, the LDH concentrations were decreased and observed between the groups treated with different concentration of CSNPs, SF-CSNPs and control (*p* < 0.05, *n* = 6). Compared with the control, the BrdU was upregulated in the groups treated with CSNPs and SF-CSNPs with significant increase (Figure 3, *p* < 0.05, *n* = 6). Those results indicate that no significant cytotoxicity was observed in the CSNPs and SF-CSNPs groups; and in addition, the CSNPs and SF-CSNPs showed improved cell growth and proliferation. Unlike metal or metal oxide nanoparticles, the CSNPs and SF-CSNPs were non-toxic. The cell growth and survival rate were increased with the higher concentration of CSNPs and SF-CSNPs. As shown in Figure 2 and Figure 3, the biocompatibility of SF-CSNPs was better than CSNPs; and the SF-CSNPs obviously enhanced HepG2 cell growth in a dose dependent manner.

### 2.4. Proteomic Analysis of Cell Response to SF-CSNPs

To investigate the effect of SF-CSNPs on normal and tumor cells of the liver, a proteomic approach, such as revised phase nano-ultra performance liquid chromatography electrospray ionization-tandem mass spectrometry (RP-nano-UPLC–ESI-MS/MS) analysis, was utilized to analyze cell lysates. The traditional methods use individual antibodies to evaluate the cell response to nanoparticles, but the proteomic approach can be used to analyze an enormous number of proteins simultaneously. In this study, HepG2 and CCL-13 cells were incubated with SF-CSNPs. After 12 h, the HepG2 and CCL-13 cells were lysed, and the cell lysates were digested by trypsin. The tryptic peptides generated were subsequently analyzed by RP-nano-UPLC–ESI-MS/MS, respectively. The fragmentation spectra obtained by the RP-nano-UPLC–ESI-MS/MS analysis in the gradient detection mode were compared with a non-redundant protein database using Mascot software. When a protein was identified by three or more unique peptides, no visual assessment of spectra was conducted and the protein was considered to be present in the sample.

In this study, more than one hundred proteins were identified and most of these were identified at the minimal confidence level, which was only one unique peptide sequence matched. Experimental results reported a total of nine protein identifications with higher confidence levels (at least three unique peptide sequences matched), and exhibited significant differences between the SF-CSNPs treated HepG2 and CCL-13 cells. A summary of the protein identifications achieved is shown in Table 2.

**Figure 4:**
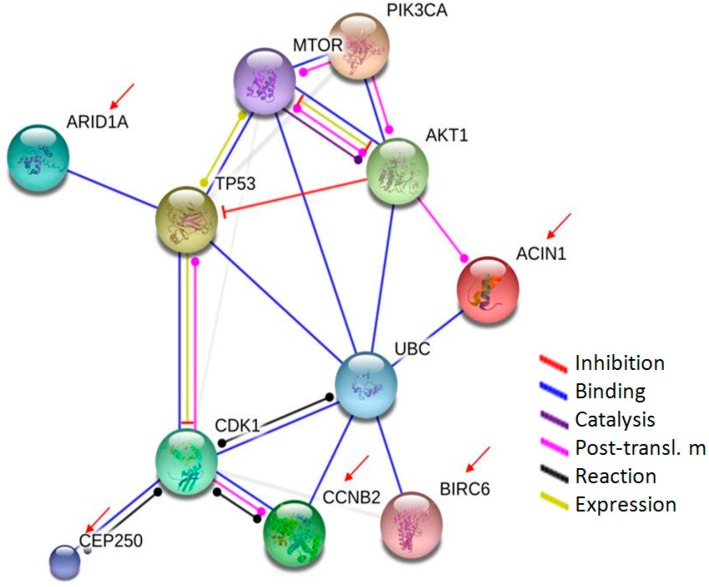
The protein–protein interaction pathways are illustrated. Proteins identified in this study are marked by arrows. The SF-CSNPs can turn on the ubiquitin pathway, which is responsible for the proliferation and is required for survival of the majority of cells.

In addition, there were five proteins identified in HepG2 cell lysate samples, which were involved in apoptosis, transcription, mitosis, cell division and cycle regulation. The five proteins with the molecular function of cell growth were positively identified as apoptotic chromatin condensation inducer in the nucleus (ACIN1, Q9UKV3), AT-rich interactive domain-containing protein 1A (ARID1A, O14497), Baculoviral IAP repeat-containing protein 6 (BIRC6, Q9NR09), G2/mitotic-specific cyclin-B2 (CCNB2, O95067), and Centrosome-associated protein CEP250 (Cep250, Q9BV73). The protein-protein interaction pathways were performed by String 9.1 Web software, and five proteins identified in this study were marked by red arrows (Figure 4). Using the protein-protein interaction pathway analysis, the main finding of SF-CSNPs treated cells is that the SF-CSNPs enhance the ubiquitin proteasome/p53 pathway in HepG2 cells which may result in tumor cell growth.

**Table 2:** The nine unique proteins identified by the higher confidence level (at least three unique peptide sequences matched) with significant difference between CCL-13 and HepG2 cells incubation with SF-CSNPs in this study.

Short Name	Swiss-Prot No.	Protein Name	MW (Da)	PI	Subcellular Location	Biological Process	Molecular Function	Peptide
SULT1A3/1A4	P50224	Sulfotransferase 1A3/1A4	34,174	5.68	Cytoplasm	Catecholamine metabolism; Lipid metabolism; Steroid metabolism	Transferase	R.LIKSHLPLALLPQTLLDQK.V; R.LIKSHLPLALLPQTLLDQK.V + Deamidated (NQ); R.LIKSHLPLALLPQTLLDQK.V + Deamidated (NQ); R.LIKSHLPLALLPQTLLDQK.V + 2 Deamidated (NQ)
ACIN1	Q9UKV3	Apoptotic chromatin condensation inducer in the nucleus	151,771	6.08	Nucleus; Nucleoplasm	Apoptosis; mRNA processing; mRNA splicing	ATPase activity; enzyme binding; nucleic acid binding; nucleotide binding; poly(A) RNA binding	R.EREMER.R; R.TSTSSSSVQAR.R + 7 Phospho (ST); K.QSADSSSSRSSSSSSSSSR.S + Deamidated (NQ); 9 Phospho (ST); K.QSADSSSSRSSSSSSSSSR.S + Deamidated (NQ); 10 Phospho (ST)
ProSAPiP1	O60299	ProSAP-interacting protein 1	71,747	7.56	Cytoplasm; Cytoskeleton			K.SRTMTPAGGSGSGLSDSGR.N + Oxidation (M); K.SRTMTPAGGSGSGLSDSGR.N + Oxidation (M); 4 Phospho (ST); R.IGTASYGSGSGGSSGGGSGYQDLGTSDSGR.A + 4 Phospho (ST); Phospho (Y); K.SRTMTPAGGSGSGLSDSGR.N + Oxidation (M); 4 Phospho (ST); K.QLQLSYVEMYQRNQQLER.R + 3 Deamidated (NQ); Phospho (Y)
PDE5A	O76074	cGMP-specific 3',5'-cyclic phosphodiesterase	99,921	5.74		blood coagulation; cGMP catabolic process; negative regulation of T cell proliferation; negative regulation of cardiac muscle contraction; positive regulation of MAP kinase activity; positive regulation of cardiac muscle hypertrophy; positive regulation of oocyte development; relaxation of cardiac muscle; signal transduction	Hydrolase	R.WILSVKKNYR.K + Phospho (ST); K.KIAATIISFMQVQK.C + Oxidation (M); Phospho (ST); K.ELNIEPTDLMNREKK.N + Deamidated (NQ); K.TQSILCMPIKNHREEVVGVAQAINK.K + 4 Deamidated (NQ); 2 Phospho (ST); R.GHTESCSCPLQQSPRADNSAPGTPTRK.I + 2 Deamidated (NQ); 5 Phospho (ST)
ARID1A	O14497	AT-rich interactive domain-containing protein 1A	241,892	6.24	Nucleus	Neurogenesis; Transcription; Transcription regulation	Chromatin regulator	K.SKKSSSSTTTNEK.I + Deamidated (NQ); 6 Phospho (ST); K.HPGLLLILGKLILLHHK.H; R.NSMTPNPGYQPSMNTSDMMGR.M + 2 Deamidated (NQ); Oxidation (M); 2 Phospho (ST); R.EMAVVLLANLAQGDSLAARAIAVQK.G + Deamidated (NQ); Oxidation (M); R.EMAVVLLANLAQGDSLAARAIAVQK.G + Deamidated (NQ); Oxidation (M); R.ITATMDDMLSTRSSTLTEDGAK.S + 2 Oxidation (M); 4 Phospho (ST); K.APGSDPFMSSGQGPNGGMGDPYSR.A + 4 Phospho (ST); Phospho (Y); R.GYMQRNPQMPQYSSPQPGSALSPR.Q + Oxidation (M); Phospho (ST); K.RNSMTPNPGYQPSMNTSDMMGR.M + Deamidated (NQ); Oxidation (M); 3 Phospho (ST); Phospho (Y)
BIRC6	Q9NR09	Baculoviral IAP repeat-containing protein 6	529,919	5.67	Golgi apparatus; Cytoplasm; Cytoskeleton	Apoptosis; Cell cycle; Cell division; Mitosis; Ubl conjugation pathway	Ligase; Protease inhibitor; Thiol protease inhibitor	K.KTSISKER.V + 2 Phospho (ST); R.YGSTNARAK.I + Deamidated (NQ); 2 Phospho (ST); R.SRGTPSGTQSSR.E + Deamidated (NQ); 3 Phospho (ST); K.MKTCVDTYTNR.L + Deamidated (NQ); Phospho (ST); Phospho (Y); R.TIPDKIGSTSGAEAANK.I + Deamidated (NQ); R.QLQDRLTPMEALLQTR.Y + Deamidated (NQ); Oxidation (M); R.GRTIPDKIGSTSGAEAANK.I + Phospho (ST); K.EKSSNVKNENTSGTR.K + 3 Deamidated (NQ); 5 Phospho (ST); K.LVNILVQLPLSGNREYSAR.V; K.WNSVFPKPGTLVQCLRLPK.F + Carbamidomethyl (C); Deamidated (NQ); K.VNYHYMSQVKNANDANSAAR.A + 5 Deamidated (NQ); Phospho (ST); R.LAQEAVTLSTSLPLSSSSSVFVR.C + Deamidated (NQ); Phospho (ST); R.GTEEICNGGMRPVVRLPSLKHQSNK.G + Carbamidomethyl (C); Deamidated (NQ); R.SFLIHVKAVNERGTEEICNGGMRPVVR.L + Deamidated (NQ); Phospho (ST)
CCNB2	O95067	G2/mitoti*C*-specific cyclin-B2	45,253	9		Cell cycle; Cell division; Mitosis	Cyclin	R.KKLQLVGITALLLASK.Y; K.VPVQPTKTTNVNKQLKPTASVKPVQMEK.L + Deamidated (NQ); Oxidation (M); Phospho (ST); K.AQNTKVPVQPTKTTNVNK.Q + 3 Deamidated (NQ); 2 Phospho (ST)
CEP250	Q9BV73	Centrosome-associated protein CEP250	280,967	5	Cytoplasm; Cytoskeleton	Cell cycle	protein *C*-terminus binding; protein kinase binding	R.EPAQLLLLLAK.T; K.GQLEVQIQTVTQAK.E + 4 Deamidated (NQ); Phospho (ST); R.QLMQERAEEGKGPSK.A + 2 Deamidated (NQ); K.ELSAQMELLRQEVK.E + Oxidation (M); Phospho (ST); R.GLHQSVRELQLTLAQK.E + Deamidated (NQ); Phospho (ST); R.DQELEALQQEQQQAQGQEER.V + 5 Deamidated (NQ); K.CVAELQKEVVLLQAQLTLERK.Q + Deamidated (NQ); K.AEHVRLSGSLLTCCLRLTVGAQSR.E; R.QEQTEVTAALAR.A + 2 Deamidated (NQ); R.RVNVELQLQGDSAQGQK.E + 2 Deamidated (NQ); Phospho (ST); K.AEHVRLSGSLLTCCLRLTVGAQSR.E; R.SLFKRGPLLTALSAEAVASALHK.L + 3 Phospho (ST)
AGBL1	Q96MI9	Cytosolic carboxypeptidase 4	120,204	6.85	Cytoplasm; Cytosol	*C*-terminal protein deglutamylation; protein side chain deglutamylation	Carboxypeptidase; Hydrolase; Metalloprotease; Protease	R.MSASFSNSTRTR.E + Deamidated (NQ); 4 Phospho (ST); K.LAPAFTMSSCSFLVEKSR.A; R.SYTMESSYCGCNQGPYQCTQR.L + Deamidated (NQ); Oxidation (M); 4 Phospho (ST); 2 Phospho (Y); R.SYTMESSYCGCNQGPYQCTQR.L + Deamidated (NQ); Oxidation (M); 4 Phospho (ST); 2 Phospho (Y); R.SYTMESSYCGCNQGPYQCTQR.L + 2 Deamidated (NQ); Oxidation (M); 4 Phospho (ST); 2 Phospho (Y); R.SYTMESSYCGCNQGPYQCTQR.L + 3 Deamidated (NQ); Oxidation (M); 4 Phospho (ST); 2 Phospho (Y); R.SYTMESSYCGCNQGPYQCTQR.L + 2 Carbamidomethyl (C); 2 Deamidated (NQ); 3 Phospho (ST); 2 Phospho (Y); R.SYTMESSYCGCNQGPYQCTQR.L + 2 Carbamidomethyl (C); 2 Deamidated (NQ); Oxidation (M); 4 Phospho (ST); Phospho (Y); R.SYTMESSYCGCNQGPYQCTQR.L + 2 Carbamidomethyl (C); 2 Deamidated (NQ); Oxidation (M); 4 Phospho (ST); Phospho (Y); R.SYTMESSYCGCNQGPYQCTQR.L + 2 Carbamidomethyl (C); 2 Deamidated (NQ); Oxidation (M); 4 Phospho (ST); Phospho (Y); R.SYTMESSYCGCNQGPYQCTQR.L + 2 Carbamidomethyl (C); 2 Deamidated (NQ); Oxidation (M); 3 Phospho (ST); 2 Phospho (Y); R.SYTMESSYCGCNQGPYQCTQR.L + 2 Carbamidomethyl (C); 2 Deamidated (NQ); Oxidation (M); 3 Phospho (ST); 2 Phospho (Y); R.SYTMESSYCGCNQGPYQCTQR.L + 2 Carbamidomethyl (C); 2 Deamidated (NQ); Oxidation (M); 3 Phospho (ST); 2 Phospho (Y); R.SYTMESSYCGCNQGPYQCTQR.L + 2 Carbamidomethyl (C); 3 Deamidated (NQ); Oxidation (M); 2 Phospho (ST); 3 Phospho (Y); R.SYTMESSYCGCNQGPYQCTQR.L + 2 Carbamidomethyl (C); 3 Deamidated (NQ); Oxidation (M); 4 Phospho (ST); Phospho (Y); R.SYTMESSYCGCNQGPYQCTQR.L + 2 Carbamidomethyl (C); 3 Deamidated (NQ); Oxidation (M); 4 Phospho (ST); Phospho (Y); R.SYTMESSYCGCNQGPYQCTQR.L + 2 Carbamidomethyl (C); Oxidation (M); 5 Phospho (ST); Phospho (Y); R.EMGVSRSYTMESSYCGCNQGPYQCTQR.L + 2 Carbamidomethyl (C); Oxidation (M); 2 Phospho (ST); 2 Phospho (Y)

**Figure 5:**
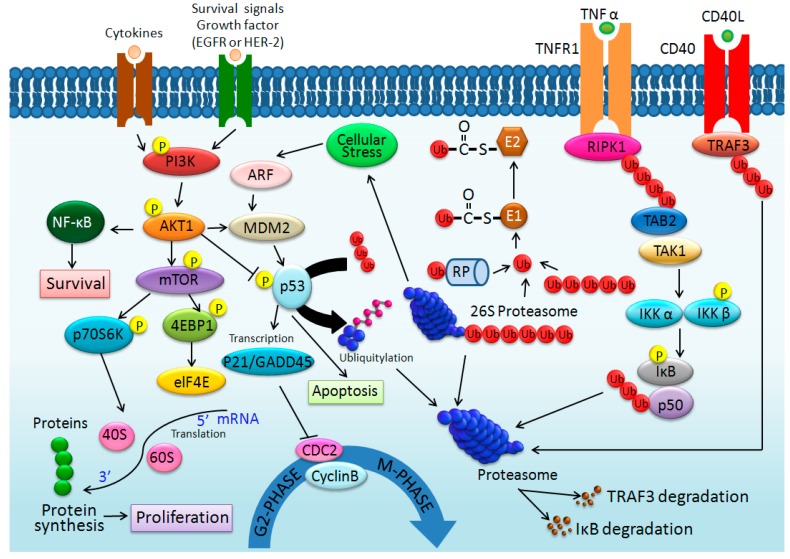
Schematic representation of some possible signaling pathways activated by SF-CSNPs which may regulate metabolism of proliferating cells through Ubiquitin C (UBC).

Ubiquitin is a 9 kDa regulatory protein that has been found in most tissues of eukaryotic organisms. There are four ubiquitin genes, *UBB*, *UBC*, *UBA52* and *RPS27A*, in the human genome. Ubiquitin is used as a covalent modifier of other proteins to activate their function and to target them for degradation, depending on the degree of ubiquitin ligation. The relevance of ubiquitination to cancer has appeared from oncogenic mutations which disrupt protein ubiquitination and control cell growth or death [23]. Ubiquitination, a post-translational modification of ubiquitin attached to substrate proteins, is carried out in three main steps: activation, conjugation, and ligation. The three steps are performed by ubiquitin-activating enzymes (E1s), ubiquitin-conjugating enzymes (E2s), and ubiquitin ligases (E3s), respectively [24]. Also, the long polyubiquitin chains are targeted for the degradation of the 26S proteasome. The polyubiquitin-26S proteasome complex may induce the p53 transcription pathway (Figure 5).

The p53 protein is also known as cellular tumor antigen p53 (UniProt name), participates in several mechanisms of anticancer function, and plays a major role in the cellular response to a wide and diverse range of stress signals, such as DNA damage, apoptosis, genomic stability, and inhibition of angiogenesis. The p53 protein is inactivated by the negative regulator, MDM2, in the cell pathway. Upon DNA damage, metabolic or cellular stresses, polyubiquitin-26S proteasome complex will lead to the dissociation of the p53 protein and MDM2 complex and the transient p53–p21 activation will promote cell survival [25]. Once activated, the p53 protein will induce cell cycle arrest to allow either repair and survival of the cell or apoptosis to discard the damaged cell [26]. The p53 protein can arrest growth by holding the cell cycle at the G2/M regulation point on DNA damage recognition. When the p53 protein holds the cell cycle, the DNA repair proteins may fix the DNA damage and the cell may go to continue the cell cycle. If DNA is under sustained damage, the p53 protein can activate DNA repair proteins and may be an important factor in aging [27].

Ubiquitin C (UBC) also participates in inactivation of mTOR, which is a master regulator of protein synthesis, translation and proliferation. The mTOR pathway may also be activated by SF-CSNP via the HER-2 receptor [28,29]. Activation of HER-2 leads to cell proliferation presumably by inducing activation of the PI3K/AKT1/mTOR pathway. The HER2/PI3K/AKT1/mTOR pathway is responsible for the proliferation, leads to a multidrug resistance in tumor cells, and is required for survival of the majority of cancer cells (Figure 5). It promotes cell growth and progression of a variety of tumors and is the most frequently deregulated signaling cascades in tumor initiation [30].

In particular, the PI3K/AKT pathway regulates the import and retention of glucose. It provides substrates for glycolysis and the biosynthetic pathways which rely on the supply of glycolytic intermediates. The mTOR pathway, downstream of AKT signaling, regulates the protein translation rate, and accelerates the supply of amino acid biosynthesis to generate the charged tRNAs [31].

In our previous study of CSNPs, six proteins, Amyloid β A4 protein (APP, P05067), C–C chemokine receptor type 8 (CCR8, P51685), CD44 antigen (CD44, P16070), G2/mitotic-specific cyclin-B2 (CCNB2, O95067), Neuroblast differentiation-associated protein (AHNAK, Q09666), and Osteonectin/SPARC protein (SPARC, P09486), were also identified in HepG-2 cell lysate samples and associated with metastasis and cell proliferation [32]. CD44 can activate the PI3K/AKT1/mTOR pathway, which is responsible for the proliferation and is required for survival of the majority of cells. The PI3K/AKT1/mTOR pathway can also be stimulated by GSK3B when GSK3B is bound with APP and SPARC. In this SF-CSNPs study, not only the PI3K/AKT1/mTOR pathway, but also the UBC/p53 pathway was activated by SF-CSNPs. In the results of LDH and proliferation tests, the SF-CSNPs were involved in cancer cell survival and proliferation with stronger influence. Due to the similar particle size and zeta potential of CSNPs and SF-CSNPs, the effect of cell proliferation may be caused by the silk fibroin coating on the surface on the CSNP.

## 3. Experimental Section

### 3.1. Silk Fibroin Nanoparticle Preparation

Chitosan nanoparticles (CSNP) were prepared according to Calvo *et al.* [33]. Briefly, water-soluble chitosan was dissolved in aqueous solution. Nanoparticles were formed spontaneously upon addition of 2 mL of the sodium tripolyphosphate (0.1% TPP in sodium citrate, 238,503, Sigma-Aldrich, St. Louis, MO, USA) aqueous solution to 5 mL of the chitosan acidic solution (448,869, Sigma-Aldrich, low molecular weight, 75%–85% deacetylation, 1 mg/mL) under magnetic stirring at room temperature. The pH of chitosan solution varied between 3.0 and 5.0. Then the PEG (10 mg/mL, 1 mL) was dissolved into the chitosan solution after the addition of the TPP solution. Nanoparticles were isolated by ultracentrifugation (25,000× *g*, 10 °C, 15 min) and then re-suspended in water by manual shaking. The CSNP (3 mg/mL) was added into the SF solution (3 mg/mL) slowly (12 mL/h) and stirred for 30 min (800 rpm). The SF-CSNPs were isolated by ultracentrifugation (25 kg, 10 °C, 15 min) and then re-suspended in water by manual shaking. The protein concentration of the SF-CSNPs was measured by Bio-Rad Bradford total protein assay kit (Bio-Rad Laboratories, Inc., Hercules, CA, USA).

### 3.2. Characterization of Silk Fibroin Nanoparticles

The particle size and size distributions of the nanoparticles were performed by the particle size analyzer (90 Plus particle sizer, Brookhaven Instruments Corp., Holtsville, NY, USA). For the particle size analysis, each sample was diluted to the appropriate concentration with filtered phosphate-buffered saline, pH = 7.4. Each analysis lasted 2 min and was performed at 25 °C with an angle detection of 90°.

Measurement of the zeta potential of nanoparticles was performed by Zeta plus 90 particle sizer (Brookhaven Instruments Corp., Holtsville, NY, USA) with a 5 mW He–Ne laser (λ = 663 nm). The zeta potential values were calculated from the mean electrophoretic mobility values using the Smoluchowski’s equation.

### 3.3. Cell Culture

HepG2 (liver tumor cell) and CCL-13 (liver normal cell) cells were maintained at 37 °C and 5% CO_2_ in RPMI 1640 medium (Gibco, Grand Island, NY, USA) supplemented with 10% fetal bovine serum (FBS, Hyclone Laboratories, Logan, UT, USA), 1% penicillin/streptomycin (Gibco, Grand Island, NY, USA) and 44 mM NaHCO_3_ (Sigma-Aldrich, St. Louis, MO, USA). After three days, the cells were washed with serum-free RPMI 1640 medium and incubated with the serum-free medium containing nanoparticles at concentrations of 1 to 5 μg/mL for 12 h.

### 3.4. Bromodeoxyuridine (5-Bromo-2'-deoxyuridine, BrdU) and Lactate Dehydrogenase (LDH) Assay

CCL-13 and HepG2 cells were seeded in a sterile 96-well tissue culture plate at 2 × 10^5^ cells/mL in 100 μL/well of appropriate cell culture media with SF-CSNPs. The cell proliferation was determined by bromodeoxyuridine assay (BrdU Cell Proliferation Assay, Millipore, Darmstadt, Germany). The cytotoxicity of SF-CSNPs was evaluated *in vitro* using the lactate dehydrogenase assay (LDH Cytotoxicity Assay, ScienCell Research Laboratories, Teaneck, NJ, USA). These assays were performed according to the manufacturers’ instructions. The absorbance values were measured by an ELISA reader (Multiskan EX, Thermo Scientific, Vantaa, Finland, reference wavelength: 450 nm).

### 3.5. Cell Morphology

For cell morphologies of HepG2 before and after incubation with SF-CSNPs, the live cell images were observed with a microscope equipped with a fluorescence light source (FLoid^®^ cell fluorescence imaging Station, Invitrogen, Grand Island, NY, USA), and the cell micrographs were taken with a charge-coupled device (CCD) camera.

### 3.6. Protein Sample Preparation

After incubation with SF-CSNPs, the HepG2 and CCL-13 cells were lysed by cell lysis buffer (3500-1, Epitomics, Inc., San Francisco, CA, USA), and cell lysates were centrifuged at 1500× *g* for 10 min at 4 °C. The supernatants were filtered by 0.8 μm filter and the protein concentrations were adjusted to 1 mg/mL by 25 mM ammonium bicarbonate.

Cell lysate samples (100 μL) were transferred into the 1.5 mL Eppendorf tubes and incubated at 37 °C for 3 h after mixing with 25 μL of 1 M dithiothreitol (DTT, USB Corporation, Cleveland, OH, USA). Then cell lysate samples were reduced and alkylated in the dark at room temperature for 30 min after addition of 25 μL of 1 M iodoacetamide (IAA, RPN6302V, Amersham Biosciences, Uppsala, Sweden) in 25 mM ammonium bicarbonate. Approximately 10 μL of 0.1 μg/μL modified trypsin digestion buffer (Trypsin Gold, Mass Spectrometry Grade, V5280, Promega, WI, USA) in 25 mM ammonium bicarbonate were added to the cell lysate samples, and the cell lysate samples were incubated at 37 °C for at least 12 h in a water bath. Two μL of formic acid was added to each sample before mass spectrometric analysis for protein identification.

### 3.7. Proteomic Analysis

The complex peptide mixtures were separated by RP-nano-UPLC–ESI-MS/MS. The protein tryptic digests were fractionated using a flow rate of 300 nL/min with a nano-UPLC system (nanoACQUITY UPLC, Waters, Milford, MA, USA) coupled to an ion trap mass spectrometer (LTQ Orbitrap Discovery Hybrid FTMS, Thermo, San Jose, CA, USA) equipped with an electrospray ionization source. For RP-nano-UPLC–ESI-MS/MS, a sample (2 μL) of the desired peptide digest was loaded into the reverse phase column (Symmetry C18, 5 μm, 180 μm × 20 mm) by an autosampler. The RP separation was performed using a linear acetonitrile gradient from 99% buffer A (100% D.I. water/0.1% formic acid) to 85% buffer B (100% acetonitrile/0.1% formic acid) in 100 min using the micropump. The separation is performed on a C18 microcapillary column (BEH C18, 1.7 μm, 75 μm × 100 mm) using the nano separation system. As peptides eluted from the micro-capillary column, they were electrosprayed into the ESI-MS/MS with the application of a distal 2.1 kV spraying voltage with heated capillary temperature of 200 °C. Each cycle of one full scan mass spectrum (*m*/*z* 400–2000) was followed by four data dependent tandem mass spectra with the collision energy set at 35%.

### 3.8. Database Search

For protein identification, Mascot software (Version 2.2.1, Matrix Science, London, UK) was used to search the Swiss-Prot human protein sequence database. Positive protein identifications were defined when the Mowse scores greater than 100 were considered significant (*p* < 0.05). Proteins were annotated by similar searches using UniProtKB/Swiss-Prot databases (SIB Swiss Institute of Bioinformatics, Lausanne, Switzerland). The protein-protein interaction pathways were performed by String 9.1 Web software (SIB Swiss Institute of Bioinformatics, Lausanne, Switzerland).

### 3.9. Statistical Analysis

All calculations used the SigmaStat statistical software (Jandel Science Corp., San Rafael, CA, USA). All statistical significances were evaluated at 95% of confidence level or better. Data are presented as mean ± standard error.

## 4. Conclusions

Previous studies have contradicting results showing whether nanoparticles are biologically compatible. In this work, a biopolymer of chitosan with silk fibroin (SF-CSNP) is introduced. CSNP was modified with silk fibroin to achieve better cell responses, and uptake by HepG2 cells was observed. Both BrdU and LDH assays showed that SF-CSNP is gentle to HepG2 cells. The same effect recurred with SF-CSNP even when the amplitude is increased. This is of interest because nanoparticles have been proposed to carry cancer drugs.

To evaluate the responses of cellular proteins induced by SF-CSNP, mass spectrometry-based proteomics is adopted to analyze complex proteins of the cell lysate and to profile proteins based on their associated cell-nanoparticle interactions. By utilizing proteomic approaches, nine proteins related to SF-CSNP were identified.

Although the ubiquitin proteasome/p53 pathways of the interactions between five proteins and SF-CSNPs were very unclear, the effects of SF-CSNPs to liver cells were sketched. In summary, SF-CSNPs may be nontoxic to HepG2 cells. Moreover, the functional groups of biomaterials may appear to be less damaging to the cells. This study proposed a new approach for the detection of proteins to assess the response of hepatic cells to a biopolymer. Knowing the response of cellular proteins induced by biomaterials may assist the development of nanoparticles. Although there was no direct evidence to prove the relevance between SF-CSNP and cell growth/mitosis pathways in our study, it is still worthwhile to be considered, especially as an anticancer drug delivery system.
